# Exosomal cancer immunotherapy is independent of MHC molecules on exosomes

**DOI:** 10.18632/oncotarget.9585

**Published:** 2016-05-25

**Authors:** Stefanie Hiltbrunner, Pia Larssen, Maria Eldh, Maria-Jose Martinez-Bravo, Arnika K. Wagner, Mikael C.I. Karlsson, Susanne Gabrielsson

**Affiliations:** ^1^ Immunology and Allergy Unit, Department of Medicine Solna, Karolinska Institute, and Karolinska University Hospital, SE-171 76 Stockholm, Sweden; ^2^ Department of Microbiology, Tumor and Cell Biology, Karolinska Institute, SE-171 77 Stockholm, Sweden

**Keywords:** exosomes, immunotherapy, MHC class I, extracellular vesicles, cancer

## Abstract

Peptide-loaded exosomes are promising cancer treatment vehicles; however, moderate T cell responses in human clinical trials indicate a need to further understand exosome-induced immunity. We previously demonstrated that antigen-loaded exosomes carry whole protein antigens and require B cells for inducing antigen-specific T cells. Therefore, we investigated the relative importance of exosomal major histocompatibility complex (MHC) class I for the induction of antigen-specific T cell responses and tumour protection. We show that ovalbumin-loaded dendritic cell-derived exosomes from MHCI^−/−^ mice induce antigen-specific T cells at the same magnitude as wild type exosomes. Furthermore, exosomes lacking MHC class I, as well as exosomes with both MHC class I and II mismatch, induced tumour infiltrating T cells and increased overall survival to the same extent as syngeneic exosomes in B16 melanoma. In conclusion, T cell responses are independent of exosomal MHC/peptide complexes if whole antigen is present. This establishes the prospective of using impersonalised exosomes, and will greatly increase the feasibility of designing exosome-based vaccines or therapeutic approaches in humans.

## INTRODUCTION

Exosomes are nano-sized membrane vesicles derived from the late endosomal compartment, capable of transferring proteins, lipids and RNA between cells [1, 2]. B cells and dendritic cells (DC) release exosomes expressing major histocompatibility complex (MHC) class I and II, as well as co-stimulatory molecules (CD80/86) and can induce peptide-specific T cell proliferation in an MHC dependent manner [3, 4]. Phase I clinical trials using DC-derived autologous exosomes as therapy have been shown to be safe but limited in inducing anti-tumour immune responses and antigen-specific T cells [5, 6].

The majority of studies investigating exosomes as a cancer vaccine or therapy have been focusing on loading exosomes with MHC class I or MHC class II restricted peptides to stimulate the immune system. However, we previously showed that only whole antigen-loaded exosomes, not peptide-loaded exosomes, are able to induce antigen-specific CD4^+^ [7] and CD8^+^ [8] T cells *in vivo*. The stimulating effect could be potentiated by adding the natural killer T (NKT) cell ligand α-galactosylceramide (αGC) onto exosomes, which significantly reduced tumour growth [9].

Interestingly, exosomes from ovalbumin (OVA)-loaded DCs carry full-length OVA as well as MHC/peptide complexes [7]. Therefore, in theory, exosomes could stimulate T cells in the recipient by three different mechanisms; i) by direct activation through the exosomal MHC/peptide complex binding to T cells [3], ii) by recycling of the exosomal MHC onto the surface of antigen presenting cells [10], or iii) by exosome degradation and full processing by recipient antigen presenting cells, with peptide loading on endogenous MHC molecules. The latter would not require an MHC match between patient and exosome donor and would greatly increase the feasibility of vesicle-based therapeutics. Therefore, we investigated if MHC on exosomes is needed to trigger T cell responses to whole OVA-loaded DC-derived exosomes *in vivo,* and if an MHC mismatch on exosomes affected their function in lymphocyte activation and tumour eradication.

Our results show that the exosome-induced immune response is independent of MHC class I expression on exosomes when delivery of whole antigen is accomplished. We demonstrate that exosomes lacking MHC class I induce OVA-specific CD8^+^ T cells and IFNγ expression to the same extent as wild type exosomes. In addition, treatment with allogeneic exosomes in a B16 melanoma model increased T cell infiltration, OVA specific antibody levels and survival, implying the possibility of using allogeneic exosomes as cancer immune therapies or vaccines.

## RESULTS

### Phenotype of B6 and MHCI^−/−^ dendritic cell-derived exosomes

**Figure 1:**
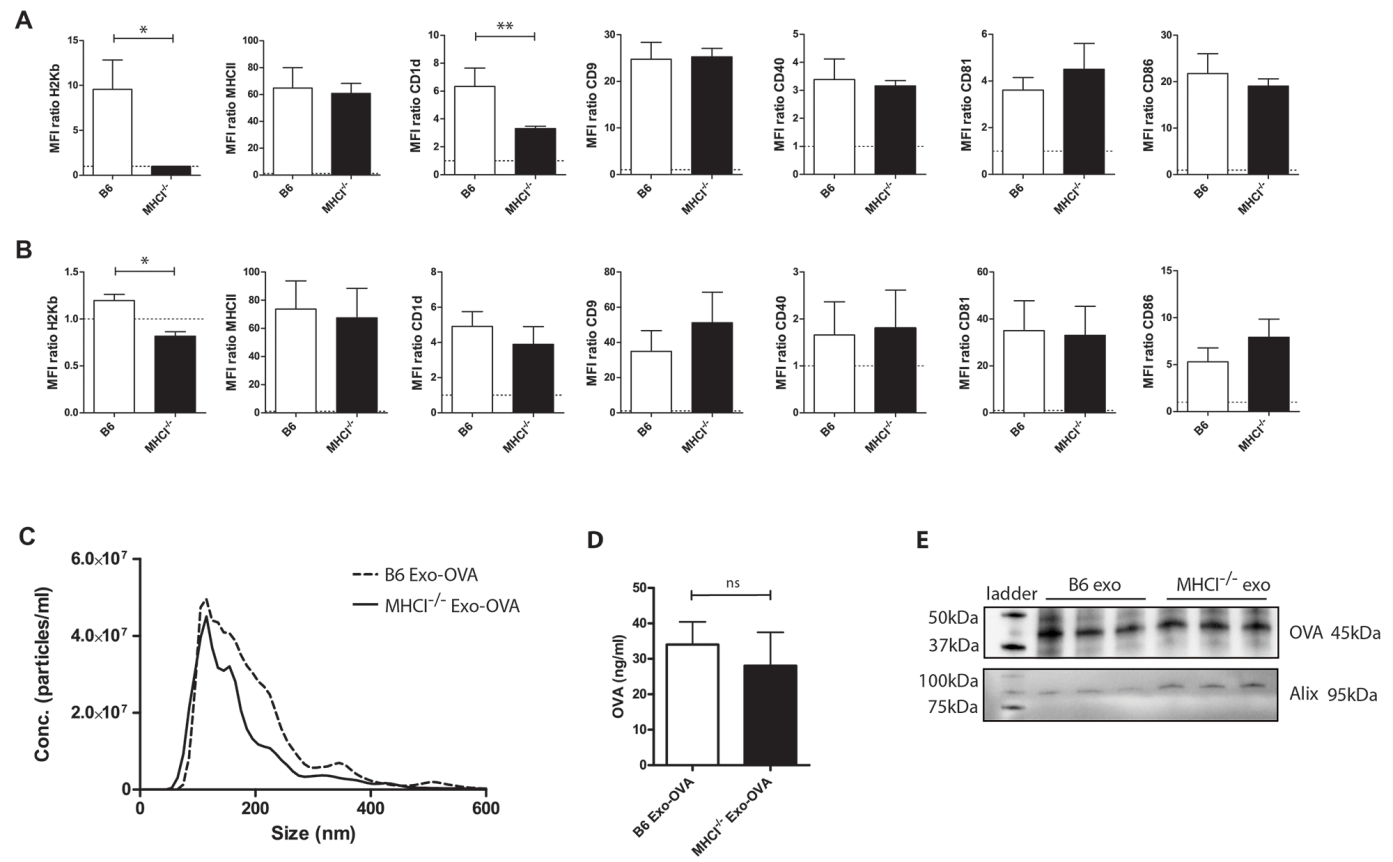
Characterization of C57Bl/6 and MHCI^−/−^ bone marrow derived dendritic cells (BMDC) and their exosomes **A.** BMDC from B6 and MHCI^−/−^ mice were analysed for surface markers by flow cytometry after 48 h of LPS activation. **B.** Exosomes from B6 and MHCI^−/−^ BMDCs were bound to anti-CD9 beads and analysed for surface markers by flow cytometry. Data in A) and B) are presented as MFI ratios between specific antibody and corresponding isotype control. **C.** Size distribution of B6 and MHCI^−/−^ exosomes measured by nanoparticle tracking analysis, data are shown as particle concentration as a mean of three different batches' mode sizes for the two types. For flow cytometry data is presented as mean ± SEM (error bars) and a non-parametric Mann-Whitney test was used, n=4-7, * P < 0.05, ** P < 0.01, **D.** Surface OVA concentrations were measured by ELISA, data represents 4 independent batches of B6 Exo-OVA and 5 independent batches of MHCI^−/−^ Exo-OVA, data represents mean ± SEM, **E.** proteins were isolated from 3 independent batches of B6 and MHCI^−/−^ exosomes and the same protein amount was analysed by western blot to compare the surface and intra exosomal amount of OVA.

First, we wanted to eliminate the possibility that exosomes from MHC class I deficient (MHCI^−/−^) DCs display a different phenotype than their wild type (WT) counterpart. Therefore, we compared expression levels of MHC class I and other immune relevant molecules on C57Bl/6 bone marrow derived dendritic cells (BMDCs) and their exosomes from WT and MHC class I^−/−^ mice. WT and MHC class I^−/−^ BMDCs and their exosomes, hereafter referred to as B6 Exo-OVA and MHCI^−/−^ Exo-OVA respectively, exhibited MHC class II (I-A/I-E), CD9, CD80, CD81, CD86, and CD40 (Figure 1A, 1B) and CD11c, CD54 and CD63 (data not shown) at similar levels. However, CD1d expression was significantly reduced on MHC class I^−/−^ BMDCs (Figure 1A) but not on their corresponding exosomes (Figure 1B). As expected, MHC class I (H2Kb) was not present on either MHC class I^−/−^ BMDCs (Figure 1A) or on their exosomes (Figure 1B). Thus, we conclude that exosomes from MHCI^−/−^ BMDCs have a similar set of costimulatory molecules as wild type exosomes. Furthermore, size distribution by nanoparticle tracking analysis (NTA) demonstrated that B6 Exo-OVA and MHCI^−/−^ Exo-OVA had a diameter of 115 and 125 nm, respectively. Exosomes could potentially carry the antigen on both their surface and internally. Therefore, OVA amounts were measured both by ELISA (Figure 1D) and western blot (Figure 1E). No differences in surface or internal OVA antigen levels were detected in B6 Exo-OVA and MHCI^−/−^ Exo-OVA. The exosome marker Alix was present at similar levels in all samples (Figure 1E).

### Exosomes induce upregulation of MHC class II expression already one hour after injection

**Figure 2:**
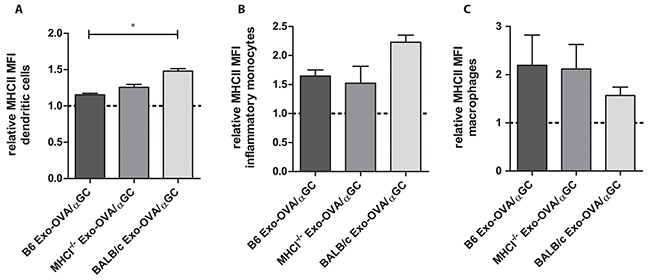
MHC class II upregulation on DC, monocytes and macrophages 1 hour after exosome injection 100 μg PKH67-labeled exosomes were injected *i.v.* into the tail vein of C57Bl/6 mice, as a control PKH67 alone was injected. **A.** MHC class II expression on CD11b^−^ CD11c+ dendritic cells, **B.** MHC class II expression on CD11b^high^CD11c^−^ Ly6C^high^Ly6G^−^F4/80^+^ inflammatory monocytes, **C.** MHC class II expression on CD11b^high^CD11c^−^ Ly6C^low^Ly6G^−^F4/80^+^ macrophages, all data are shown as MFI ratios towards the dye control and as mean ± SEM. Data were analysed by Kruskal-Wallis with Dunn's multiple comparisons, * P < 0.05.

To test whether exosomes activate and target antigen presenting cells (APC) in the spleen, we injected PKH67 stained Exo-OVA/αGC B6, MHCI^−/−^ and BALB/c *i.v.* and analysed MHC class II expression on APCs in the spleen one hour after injection. The PKH67 signal was hardly detected, therefore only MHCII expression on recipient cells was analysed. DCs, inflammatory monocytes and macrophages upregulated MHCII expression already one hour after injection compared to a dye control (Figure 2). No difference in MHCII expression was seen on B cells. However, we have previously seen that Exo-OVA/αGC induce upregulation of CD69 on B cells already 24h after injection (unpublished data). We conclude that exosomes stimulate DCs, macrophages and B cells in the spleen early after injection.

### Induction of CD8^+^ T cells is independent of exosomal MHC class I

**Figure 3:**
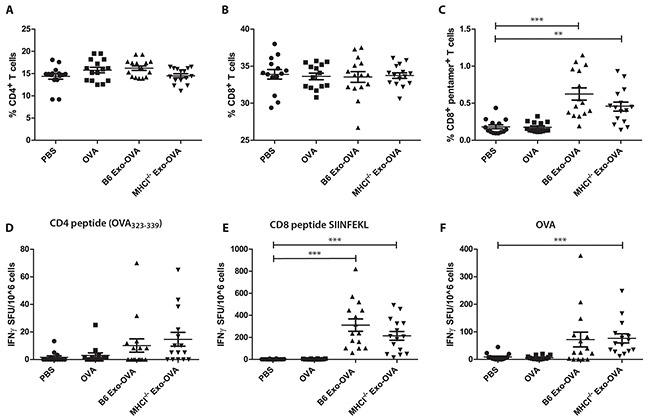
WT B6 and MHCI^−/−^ exosomes induce similar percentages of OVA specific CD8^+^ T cells 40 μg of B6 Exo-OVA, MHCI^−/−^ Exo-OVA, soluble OVA (300 ng) or PBS were injected *i.v*. into B6 mice, which were sacrificed and analysed 7 days after injection. Percentage (%) of total **A.** CD4^+^ and **B.** CD8^+^ T cells **C.** OVA specific T cells (defined as live cells, B220^−^, TCRβ^+^, CD8^+^, pentamer^+^). IFNγ ELISPOT after *ex vivo* restimulation for 19 h with **D.** CD4 peptide OVA_323-339_ **E.** CD8 peptide SIINFEKL or **F.** whole OVA. Data are pooled from 3 independent experiments, n=14-15. Dots represent a single mouse and data are presented as mean ± SEM. Data were analysed by Kruskal-Wallis with Dunn's multiple comparisons, * P < 0.05, ** P < 0.01, *** P < 0.001.

The induction of antigen-specific T cells is a major goal in cancer immunotherapy. However, we have shown the efficacy of protein loaded over peptide-loaded exosomes *in vivo*. Therefore, we asked whether MHC class I molecules on exosomes are needed for the induction of antigen-specific T cells. We injected 40 μg of B6 Exo-OVA, MHCI^−/−^ Exo-OVA, free OVA (300 ng) or PBS *i.v.* into C57Bl/6 recipient mice and analysed the immune response 7 days after injection. The percentage of total splenic CD4^+^ T cells (Figure 3A) and CD8^+^ T cells (Figure 3B) were equal in all treatment groups. OVA specific CD8^+^ T cells were significantly increased in B6 Exo-OVA and MHCI^−/−^ Exo-OVA injected groups compared to PBS and soluble OVA with no difference between the exosome injected groups (Figure 3C). In addition, *in vitro* CFSE proliferation assay with OT-I/Rag2^−/−^ splenocytes (specific transgene that encodes a T cell receptor specific for SIINFEKL peptide) showed similar proliferation after stimulation with B6 Exo-OVA or MHCI^−/−^ Exo-OVA (Supplementary Figure S1). Importantly, free OVA was not immunostimmulatory, demonstrating the adjuvant effect of the exosomes (Figure 3C). After *ex vivo* restimulation with the CD4-restricted peptide (OVA_323-339_), no significant differences in IFNγ production between the groups were observed (Figure 3D). In line with the flow cytometry data (Figure 3C), restimulation with SIINFEKL or OVA induced similar levels of IFNγ producing cells in B6 Exo-OVA or MHCI^−/−^ Exo-OVA mice (Figure 3E, F). Taken together, these data support the hypothesis that MHC class I is not needed on the exosomes for inducing an antigen-specific CD8^+^ T cell response.

### OVA/αGC exosomes enhance CD8^+^ T cell stimulation independently of MHC class I

**Figure 4:**
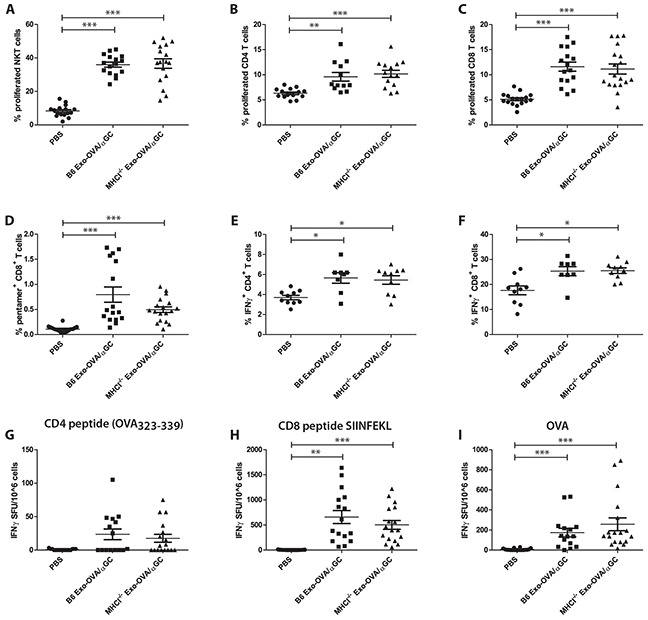
Exosomes from WT B6 or MHCI^−/−^ mice loaded with αGC and OVA induce similar immune responses B6 mice were injected with 40 μg B6 Exo-OVA/αGC or MHCI^−/−^ Exo-OVA/αGC and fed for 7 days with BrdU to assess *in vivo* proliferation. On day 7 mice were sacrificed and splenocytes were analysed by flow cytometry. **A.** Splenic NKT cell proliferation (defined as live cells, B220^−^, TCRβ, NK1.1^+^, DimerX^+^, BrdU^+^) **B.** CD4^+^ T cell proliferation **C.** CD8^+^ T cell proliferation **D.** percentage (%) of OVA specific T cells (defined as live cells, B220^−^, TCRb^+^, CD8^+^, pentamer^+^). Intracellular flow cytometry analysis for IFNγ of **E.** CD4^+^ T cells and **F.** CD8^+^ T cells after 4 h restimulation with PMA/Ionomycin/Brefeldin A. Data are pooled from two independent experiments, n=8-10. IFNγ ELISPOT of mice injected with either B6 Exo-OVA or MHCI^−/−^ Exo-OVA after *ex vivo* restimulation for 19 h with **G.** CD4 peptide OVA_323-339_, **H.** CD8 peptide SIINFEKL or **I.** whole OVA. Data are pooled from 4 independent experiments, n=16-18. Dots represent a single mouse and data are presented as mean ± SEM. Data were analysed by Kruskal-Wallis with Dunn's multiple comparisons, * P < 0.05, ** P < 0.01, *** P < 0.001.

Next, we investigated if potentiating the immune system by adding αGC onto exosomes would influence the independence of MHC class I molecules on exosomes. We have previously described that αGC associated with exosomes synergistically stimulates OVA specific CD4^+^ and CD8^+^ T cells and reduces tumour growth significantly, compared to only OVA-loaded exosomes [9]. Exosomes from B6 and MHCI^−/−^ BMDCs induced similar levels of NKT cell proliferation (Figure 4A), indicating similar levels of αGC on the two exosome types. No differences between B6 and MHCI^−/−^ exosome induced CD4^+^ and CD8^+^ T cell proliferation (Figure 4B, C) and percentages of OVA-specific CD8^+^ T cells (Figure 4D) were detected. Exosomes did not induce any changes in T follicular helper cells or percentages of marginal zone B cells, however they induced a slight increase in follicular B cells and germinal center B cells, but with no differences between B6 and MHCI^−/−^ exosomes (data not shown). We further restimulated splenocytes with PMA/Ionomycin/Brefeldin A and determined intracellular IFNγ expression. Both exosome types led to a similar increase in IFNγ^+^CD4^+^ (Figure 4E) and IFNγ^+^CD8^+^ (Figure 4F) T cells. ELISPOT assay showed that restimulation with SIINFEKL peptide (Figure 4H) and whole OVA (Figure 4I) induced comparable numbers of IFNγ expressing cells. No significant effect was observed when splenocytes were restimulated with CD4 peptide (Figure 4G). Importantly, B6 Exo-OVA/αGC and MHCI^−/−^ Exo-OVA/αGC exosomes did not show any differences in their capacity to induce IFNγ.

### Cancer therapy is independent of exosomal MHC molecules

**Figure 5:**
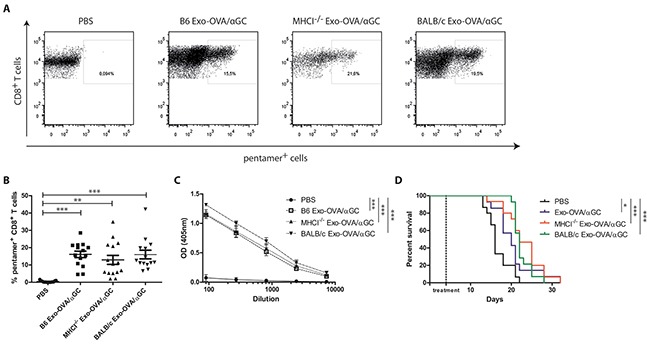
OVA specific CD8^+^ T cell induction in B16 tumours by exosomes is independent of MHC molecules on exosomes B6 mice were injected *s.c.* with 200,000 B16/OVA melanoma cells, tumour growth was monitored and mice were treated *i.v.* with 40 μg B6 Exo-OVA/αGC, MHCI^−/−^ Exo-OVA/αGC or BALB/c Exo-OVA/αGC 4 days after tumour injection. Mice were sacrificed when the tumour reached a volume of 1,000 mm^3^. **A.** Representative flow cytometry plots (presented as CD45^+^, B220^−^, TCRb^+^, CD8^+^, pentamer^+^ cells) and **B.** analysis of OVA specific CD8^+^ T cells infiltrated into the tumour of all four treatment groups. Dots represent a single mouse and data are presented as mean ± SEM. Data were analysed by Kruskal-Wallis with Dunn's multiple comparisons **C.** OVA-specific IgG antibodies in the sera of the sacrificed animals was determined by ELISA, **D.** Kaplan-Meier survival curve, data were analysed by Mantel-Cox test. Data represent 2 independent experiments, n=14-15, * P < 0.05, ** P < 0.01, *** P < 0.001.

We wanted to investigate if exosomes lacking MHC class I are capable of diminishing tumour growth in a therapeutic tumour model. With the goal to increase feasibility of future cancer treatments in humans, we also tested allogeneic exosomes, i.e. with an MHC mismatch in both MHC class I and II (BALB/c Exo-OVA/αGC). BALB/c exosomes expressed a similar phenotype compared to B6 and MHCI^−/−^ and had similar OVA levels (Supplementary Figure S2A, S2B, S2C). Furthermore, they were able to induce *in vitro* proliferation of splenocytes from OTI/RAG2^−/−^ in comparable levels to B6 and MHCI^−/−^ exosomes (data not shown). B6 mice were injected *s.c.* with 200,000 B16/OVA F1 melanoma cells followed by treatment with 40 μg of B6 Exo-OVA/αGC, MHCI^−/−^ Exo-OVA/αGC or BALB/c Exo-OVA/αGC four days after tumour inoculation. Tumour bearing mice showed enhanced tumour-infiltrating T cells and OVA specific T cells (Figure 5A and 5B, Supplementary Figure S3) and increased overall survival (Figure 5D) in all exosome treated groups. No significant difference was observed in tumour-infiltrating T cells between B6 Exo-OVA/αGC, MHCI^−/−^ Exo-OVA/αGC and BALB/c Exo-OVA/αGC treated mice (Figure 5B). This suggests that MHC molecules on exosomes are not needed to trigger anti-tumour immune responses *in vivo*. Furthermore, serum levels of OVA-specific antibodies were similar in all treatment groups (Figure 5C), which indicates that also the B cell response is independent of MHC on exosomes.

To see whether a delayed model would separate the groups more, we reduced the number of injected tumour cells. However, also when injecting 30,000 B16/OVA melanoma cells, a significant increase in survival was seen in all treated groups, with the strongest effect seen after injecting BALB/c exosomes (Supplementary Figure S4A and S4B). Importantly, the MHC mismatch did not negatively influence the antigen-specific anti-tumour response compared to syngeneic or MHCI^−/−^ exosomes.

## DISCUSSION

The discovery of MHC/peptide complexes on exosomes led to the idea of using DC-derived exosomes to stimulate antigen-specific T cells in immunotherapy against cancer [11]. Indeed, peptide/MHC complexes on exosomes have been shown to be capable of stimulating T cells [10], however, treatment with peptide-loaded exosomes has not been as efficient as anticipated [5, 6]. We have shown that whole antigen is carried by exosomes derived from antigen-pulsed DCs. Therefore, we questioned the relative importance of delivery of MHC/peptide complexes versus whole antigen in eliciting immune responses *in vivo*. In this study, we demonstrate that MHC molecules on exosomes are not crucial for inducing T cell responses or anti-tumour immunity, thereby negating the need for autologous DCs in exosome-based immunotherapy.

Here, we have used exosomes loaded with whole antigen and not only peptides. As exosomes and their cargo are easily taken up and processed by DCs [12], the antigenic peptides from the delivered antigen can be presented on MHC on the recipient DCs. Indeed, we show that exosomes activate DCs, monocytes and macrophages already one hour after injection (Figure 2). Furthermore, DCs have previously been shown to be important for a response to exosomes, as injection of OVA-pulsed DC-derived exosomes induce CD8^+^ T cell responses *in vivo* only in the presence of CD11c expressing cells in the marginal zone [13, 14]. Interestingly, even adoptively transferred peptide-loaded DC need endogenous antigen-presenting cells to stimulate T cells [15]. In addition, Morelli et al have shown that exosomal antigens can be cross-presented to CD8^+^ T cells by DCs [12].

The use of the patients' own DC-derived exosomes in therapy is complicated, as it is not optimal to perform leukapheresis on already immunocompromised patients. Therefore, an evaluation of the possibility of using MHC mismatched exosomes is crucial for designing future exosome-based vaccines. In this study, we show that allogeneic and syngeneic exosomes stimulate similar percentages of tumour-infiltrating T cells in a B16 mouse melanoma model. This challenges the current dogma of the central role for MHC class I on exosomes to stimulate T cell responses. Our results demonstrate the potential of using non-personalised exosomes as cancer treatment, where allogeneic exosomes could be loaded with lysate from the patient's own cancer cells. Whether other antigens can be loaded in exosomes as easily as OVA needs to be verified, but indeed, the antigen loading onto DC-derived exosomes could also be accomplished with hen egg white lysozyme (HEL) (our own unpublished data). It has been demonstrated that OVA uptake by DC is mainly mediated through the mannose receptor (MR), while cell associated-OVA is taken up MR independently [16]. However, different receptors have been shown to be crucial for exosome binding and uptake which questions the necessity of MR receptors in our study [17].

One crucial point to consider if using allogeneic exosomes in cancer therapy is, whether the patient's own DCs will be able to express the required amount of MHC on exosomes to induce a response. This might be overcome by the delivery of additional potentiating signals in or on the exosomes. Other immunostimulatory molecules like TLR ligands will induce MHC expression on the DCs and indeed have been shown to further potentiate the response to exosomes [18]. Previously, we published that co-delivery of the NKT cell ligand αGC and OVA on exosomes potentiate the immune response without inducing anergy after two injections and that these exosomes reduced tumour growth in the B16/OVA mouse melanoma model [9]. Here, we showed that the boosting effect of αGC also is independent of MHC.

Compared to allogeneic transplantation of organs where the antigens are constantly present, exosomes are quickly cleared from the blood in the marginal zone and are mainly taken up by macrophages [19] or DCs in the marginal zone [12]. It has previously been shown that DCs can present exosomal allo-peptides to T cells and induce T cell proliferation [12]. In cancer therapy this might even be beneficial. MHC specific antibodies binding after the second injection might lead to the formation of immune complexes which can induce higher antibody titers compared to the soluble antigen [20]. In addition, immune complexes induce more cross-presentation and subsequently more antigen-specific CD8^+^ T cells compared to soluble antigen alone [21]. The potent B cell stimulation and antigen-specific antibody production induced by both syngeneic and allogeneic exosomes further supports the use of allogeneic exosomes in vaccine approaches.

**Figure 6:**
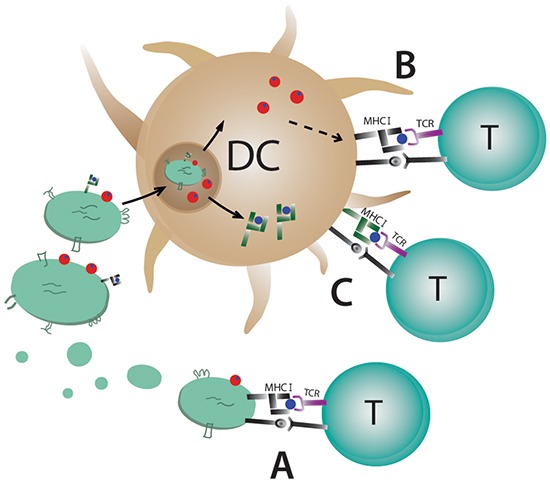
Possible mechanisms for T cell stimulation by antigen-loaded exosomes Dendritic cell-derived exosomes carry MHC class I and II molecules on the surface and **A.** can stimulate T cells directly by binding to the T cell receptor. Direct stimulation of T cells requires syngeneic MHC (black) molecules on exosomes due to MHC restriction. **B.** Uptake of exosomes by antigen presenting cells can lead to their degradation and loading of exosomal peptides on the APC's MHC molecules or **C.** the whole exosomal MHC/peptide complex (green) can be recycled and exposed on the cell surface.

In summary, we have shown that MHC class I molecules on exosomes are not needed for eliciting an antigen-specific immune response if the whole antigen is present. Furthermore, we have demonstrated that even allogeneic exosomes are able to induce an anti-tumour immune response *in vivo*. We conclude that direct T cell stimulation by exosomes *in vivo* is negligible when whole antigen is present and that DC are taking up syngeneic and allogeneic antigen-loaded exosomes, process them and induce an antigen-specific T and B cell response (Figure 6B). This offers new possibilities in designing exosomal based cancer vaccines or treatments for other diseases, and hence provides new opportunities in using allogenic DC-derived exosomes in patients.

## MATERIALS AND METHODS

### Mice

C57Bl/6 (Taconic, Tornbjerg, Denmark), BALB/c (Taconic, Tornbjerg, Denmark) or (Charles River, Sulzfeld, Germany), OT-I/RAG2^−/−^, C57Bl/6 MHCI^−/−^ (H2Kb and H2Db gene knockout) (kindly donated by Prof. Klas Kärre, Karolinska Institutet) were under specific pathogen-free conditions at the Karolinska Institutet's animal facility. The Stockholm Regional Ethics Committee approved all experiments.

### Bone marrow derived dendritic cells

Bone marrow derived dendritic cells (BMDCs) from 6 to 8 weeks old female C57Bl/6, MHCI^−/−^ on a C57Bl/6 background, and BALB/c mice were cultured with GM-CSF (Ag8653/X63 clone) and IL-4 (2 ng/ml) as previously described [7]. On day 6, 300 μg/ml Ovalbumin (Sigma) and where indicated, 100 ng/ml α-galacosylceramide (αGC) (KRN-7,000; Laradon Fine Chemicals) were added over night. On day 7, the antigens were washed away and the cells were cultured for 48 hours in media containing exosome-depleted FCS [22], GM-CSF, IL-4 and 30 ng/ml LPS (Sigma).

### Exosome preparation from BMDC supernatant

Supernatant from BMDC culture (day 9) was centrifuged for 10 min at 300 g to remove cells, supernatant was transferred and centrifuged again for 30 min at 3,000 g to remove cell debris and subsequently filtered through 0.22 μm pore sized filters (Nordic Biosite). Exosomes were isolated by ultracentrifugation for 2 hours at 100,000 g (Optima LXP-100, Beckmann Coulter). After one washing step with PBS the exosome enriched pellet was resuspended in a small volume of PBS, protein was measured by using DC protein assay (Biorad) according to the manufacturer's protocol, subsequently exosomes were stored at −80°C until further use.

### Phenotypic analysis of exosomes by FACS

10 μl Sulfate-aldehyde latex microsphere beads (4 μm, 1.3 × 10^9^ beads/ml, Invitrogen) were incubated with 10 μg anti-mouse CD9 antibody (KMC8, BD Pharmingen) for 30 min at RT. The volume was adjusted to 500 μl and rotated over night at RT. After washing, beads were blocked with 100 mM glycine for 30 min followed by washing with 0.5% BSA/PBS. Exosomes were bound to anti-CD9 coated beads with a concentration of 2 μg exosomes per μl beads and phenotyped as described previously [7]. The exosome-bead complexes were analysed on a FACS Calibur (BD Bioscience) by FlowJo software (Tree Star Inc.).

### Nanoparticle tracking analysis

The exosome size was measured by Nanoparticle tracking analysis (LM10HSB system, NanoSight, Amesbury, U.K.) equipped with a 405 nm laser running an NTA 3.0 analytical software package. Exosomes were diluted in PBS to reach a concentration of around 45 particles/frame and within 2 × 10^8^ to 8 × 10^8^ particles/ml. Three independent exosome batches were run 5 times each for 60 sec with a camera level of 10 and screen gain of 2.6 with a syringe pump speed of 50.

### In vitro proliferation

Single cell suspension of spleen and lymph nodes from 6-8 week old OT-I/RAG2^−/−^ mice was prepared by using 70 μm cell strainers (BD Biosciences). Red blood cells were lysed with ACK buffer (0.8% NH_4_Cl, 0.1% KHCO_3_, 0.1 mM EDTA [pH 7.3]) and washed with complete media (RPMI 1640 medium supplemented with 200 mM L-glutamine, 100 IU/ml penicillin-streptomycin (Thermo Scientific), and 10% FCS (HyClone, Thermo Scientific) followed by a wash with PBS. Cells were stained with 5 μM CFSE according to the manufacturer's protocol (Life Technologies) and incubated for 10 min at 37°C. The reaction was stopped by using complete media and cells were plated out at 2 × 10^5^ cells per well in a 96 well u-bottom plate. C57Bl/6, MHCI^−/−^ or BALB/c exosomes were added in two different concentrations (1 μg or 10 μg per well), the OVA peptide SIINFEKL (2 μg/ml) was used as a positive control. The plate was incubated for 5 days at 37°C, 5% CO_2_. On day 5 cells were stained for CD8, TCRβ and CD44. Cells were analysed by a BD LSR Fortessa III (BD Bioscience) and FlowJo software (Tree Star Inc.).

### Analysis of MHC class II expression in spleen 1h after exosome injection

Exosomes from C57Bl/6, MHCI^−/−^ and BALB/c mice were stained with PKH67 (Sigma) as described before [23] and injected *i.v*. into C57Bl/6 mice. Mice were sacrificed one hour after injection and spleen was taken. Dye only was injected as a control. Dendritic cells were defined to be CD11b^−^ CD11c^+^, inflammatory monocytes CD11b^high^CD11c^−^ Ly6C^high^Ly6G^−^F4/80^+^ and macrophages CD11b^high^CD11c^−^ Ly6C^low^Ly6G^−^F4/80^+^. Cells were analysed by a BD LSR Fortessa III (BD Bioscience) and FlowJo software. All used antibodies (1:400 dilution) are listed in the Supplementary Data (Supplementary Table S1).

### In vivo proliferation

6-8 week old female C57Bl/6 mice were injected *i.v.* with 40 μg exosomes in 100 μl PBS or with the corresponding amount of soluble ovalbumin (OVA) (300 ng/mouse) on day 0. Mice were fed with 0.8 mg/ml 5-bromo-2′-deoxyuridine (BrdU, Sigma) in drinking water supplemented with 2.5% sugar for 7 days. On day 7 mice were sacrificed and blood and spleens were collected. Single-cell suspensions from the splenocytes were prepared as previously described [7] and serum was collected from coagulated blood and frozen at −20°C. Splenocytes were stained and BrdU incorporation was measured according to the manufacturer's protocol (BrdU staining kit, BD Bioscience). SIINFEKL positive CD8^+^ T cells were analysed by using the (PE)-labeled H-2Kb/SIINFEKL pentamer (ProImmune). NKT cells were analysed using the CD1d:Ig Recombinant Fusion Protein (DimerX; BD Bioscience). Cells were analysed by a BD LSR Fortessa III (BD Bioscience) and FlowJo software. All used antibodies (1:400 dilution) are listed in the Supplementary Data (Supplementary Table S1).

### Intracellular cytokine staining

Single cell suspensions of splenocytes were stimulated *ex vivo* for 4 hours with 50 ng/ml Phorbol-12-myristate 13-acetate (PMA), 500 ng/ml Ionomycin and 1 μg/ml Brefeldin A (all from Sigma) in complete media. Intracellular cytokine staining for IFNγ (Biolegend) was performed by using transcription factor staining set (eBioscience), according to the manufacturer's protocol.

### Enzyme-linked immunospot assay

IFNγ specific ELISPOT was performed according to the manufacturer's instructions (Mabtech). 200,000 splenocytes per well were plated out on PVDF membrane plates (Millipore) followed by stimulation for 19 hours in 37°C with 2 μg/ml SIINFEKL, 2 μg/ml OVA_323-339_ peptide (Innovagen), 2 μg/ml Concanavalin A (Sigma), 2 μg/ml αGC, or culture media as a control.

### Western blot

Total proteins were extracted from purified exosomes using RIPA buffer, sonication and vortexing. The exosomal proteins (10 μg) were run on a Mini-Protean TGX precast gel (Any kD, Bio-Rad Laboratories, Hercules, CA, USA) and blotted to Trans-Blot Mini PVDF membranes using the Trans-Blot Turbo™ Transfer system (Bio-Rad). Blots were incubated with blocking buffer (5% non-fat milk/PBST) overnight at 4°C. OVA was detected using an anti-OVA antibody (1:1,000; 0220-1682, AbD Serotec, Kidlington, UK), together with a donkey-anti-rabbit secondary antibody (1:10,000; NA9340, GE Healthcare, Little Chalfont, UK). Alix was detected using an anti-Alix antibody (1:1000; 2171 (3A9), Cell Signaling technology, Danvers MA, US), together with a sheep-anti-mouse secondary antibody (1:2000; NA9310V, GE Healthcare, Little Chalfont, UK) and visualized by enhanced chemiluminescence (GE Healthcare), the ChemiDoc™ MP Imaging System and Image Lab™ software version 4.1 (both from Bio-Rad).

### ELISA

The OVA concentrations on the surface of exosomes were determined by ELISA. 10 μg/ml exosomes were coated on ELISA plates over night at 4°C. Mouse anti-OVA antibody (Nordic Biosite, 1:10,000) was added and incubated for 2 hours at RT, followed by anti-mouse IgG HRP (Southern Biotech, 1:2,000) as secondary antibody incubated for 1 hour at RT, 3,3′,5,5′-Tetramethylbenzidine (TMB) substrate was used for detection according to the manufacturer's protocol (Biolegend), the reaction was stopped by using 1 M H_2_SO_4_. The plates were read at 450 nm using an ELISA reader (Perkin Elmer, Enspire 2300 Multilabel reader). OVA specific IgG antibody levels were determined as described before [7].

### B16/OVA melanoma tumour model

200,000 or 30,000 B16/OVA melanoma cells (F1) were injected *s.c.* in the right flank of C57Bl/6 mice. On day 4 mice were injected *i.v.* with either 100 μl PBS as a control or 40 μg of exosomes. Tumour growth was monitored and mice were sacrificed when the tumour volume reached 1,000 mm^3^. The tumour was cut into small pieces and incubated with Collagenase/Hyaluronidase (Roche) for 30 min at 37°C. Subsequently, the tissue was passed through a 100 μm cell strainer and the single cell suspension was stained for FACS analysis using antibodies against CD45, TCR-β, CD8 (Supplementary Table S1) and PE-labeled H-2K^b^/SIINFEKL pentamer (ProImmune).

### Statistical analysis

Non-parametric data was analysed by Kruskal-Wallis with Dunn's correction. All analyses were done by using GraphPad software version 6.0 (GraphPad Inc.).

## SUPPLEMENTARY FIGURES AND TABLE




