# Supplementary material for: Exosomal cancer immunotherapy is independent of MHC molecules on exosomes
Source: Oncotarget. 2016 May 25;7(25):38707–17. doi: 10.18632/oncotarget.9585 (PMC5122422; doi:10.18632/oncotarget.9585)
Supplement: Supplementary file 1 [file oncotarget-07-38707-s001.pdf]

Exosomal cancer immunotherapy is independent of MHC molecules on exosomes

SUPPLEMENTARY FIGURES AND TABLE

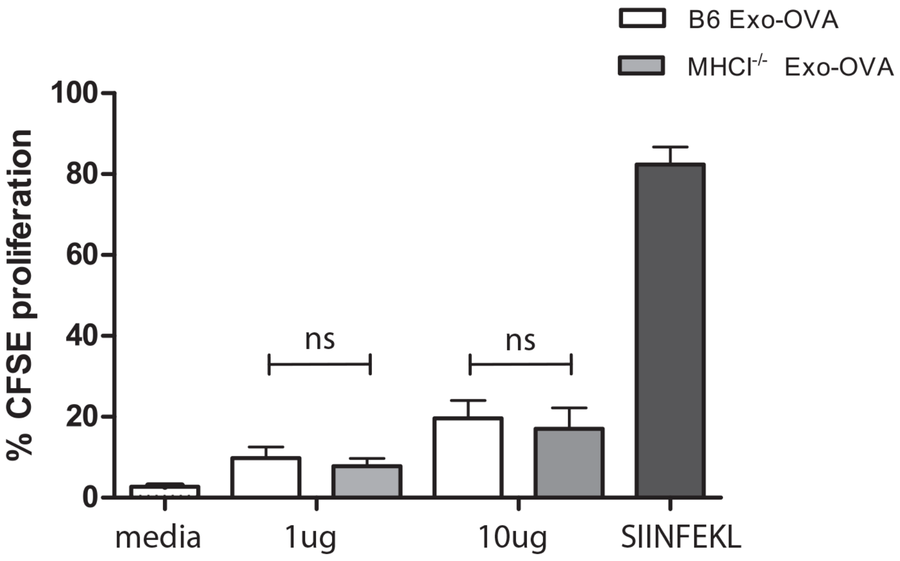

Supplementary Figure S1: B6 Exo-OVA and MHCI$^{-/-}$ Exo-OVA induce similar CD8^{+} T cell proliferation *in vitro*. To investigate the capacity of OVA loaded exosomes (B6 and MHCI$^{-/-}$) to induce T cell proliferation *in vitro*, spleen and lymph node cells from OT-I/RAG2$^{-/-}$ mice were stained with CFSE and cultured in a 96 well plate with different exosome concentrations (1 µg, 10 µg per well) for five days. B6 and MHCI$^{-/-}$ exosomes loaded with OVA induced proliferation in a concentration dependent manner significantly different from the media control (B6 Exo-OVA vs. media $P < 0.01$, MHCI$^{-/-}$ Exo-OVA vs. media $P < 0.05$, for both exosome concentrations). Data is shown as mean ± SEM. Data were analysed by Kruskal-Wallis with Dunn´s multiple comparisons, n=8-9.

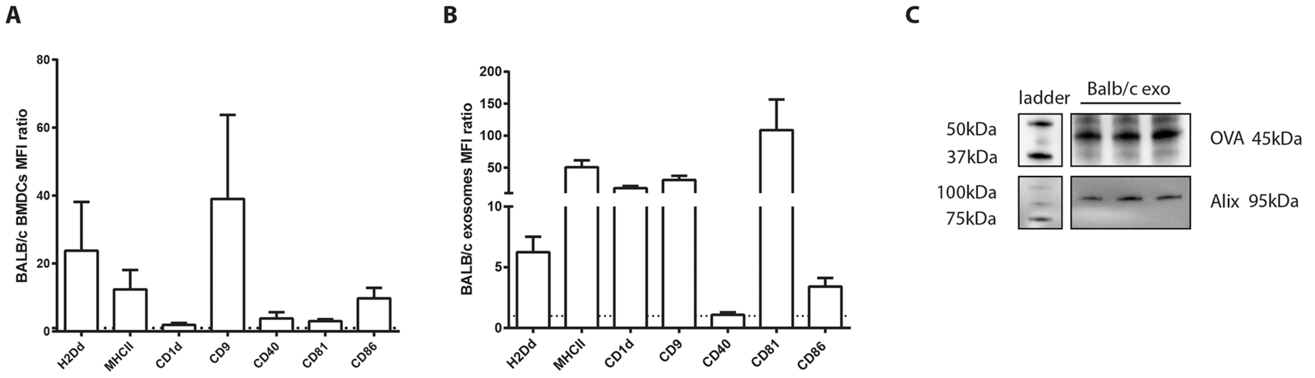

Supplementary Figure S2: Characterization of BALB/c BMDC and corresponding exosomes A. BMDCs from BALB/c mice were analysed for surface markers by flow cytometry after 48 h of LPS activation. **B.** Exosomes from BMDC of BALB/c mice were bound to anti-CD9 beads and analysed for surface markers by flow cytometry. Data in A) and B) are presented as MFI ratios between specific antibody and corresponding isotype control. **C.** Western blot analysis of the OVA content and Alix in BALB/c exosomes, data show results from two different preparations. Flow cytometry data is presented as mean ± SEM (error bars), n=4-7.

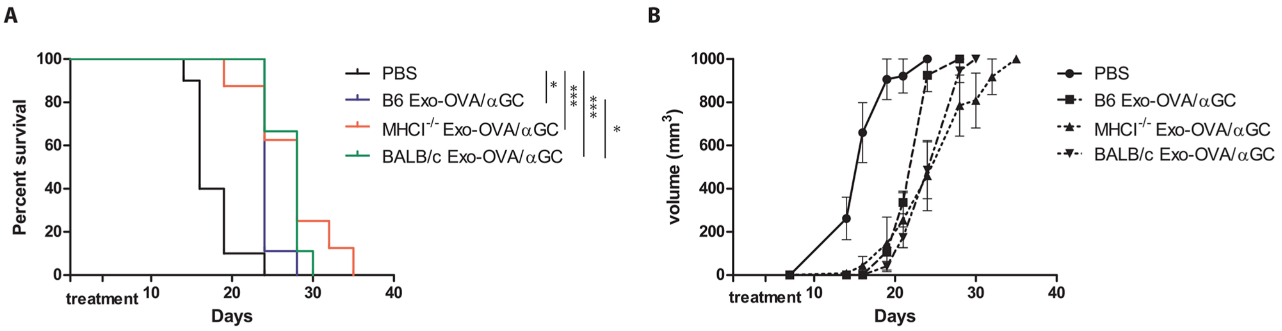

Supplementary Figure S3: Treatment of B16/OVA melanoma tumours with B6, MHCI$^{-/-}$ or BALB/c Exo-OVA/αGC prolongs survival similarly also when lower numbers of tumour cells are injected. B6 mice were injected *s.c.* in the right flank with 30 000 B16/OVA cells and treated with 40 µg Exo-OVA/αGC of B6, MHCI$^{-/-}$ or BALB/c mice 4 days after tumour inoculation. Mice were sacrificed when the tumour reached a volume of 1000 mm^3. **A.** Treatment with exosomes prolonged survival in all groups significantly compared to PBS control and **B.** also reduced tumour volume. Kaplan-Meier survival curve, data was analysed by Mantel-Cox test. Data represent one experiment, n=8-10, * $P < 0.05$, ** $P < 0.01$, *** $P < 0.001$.

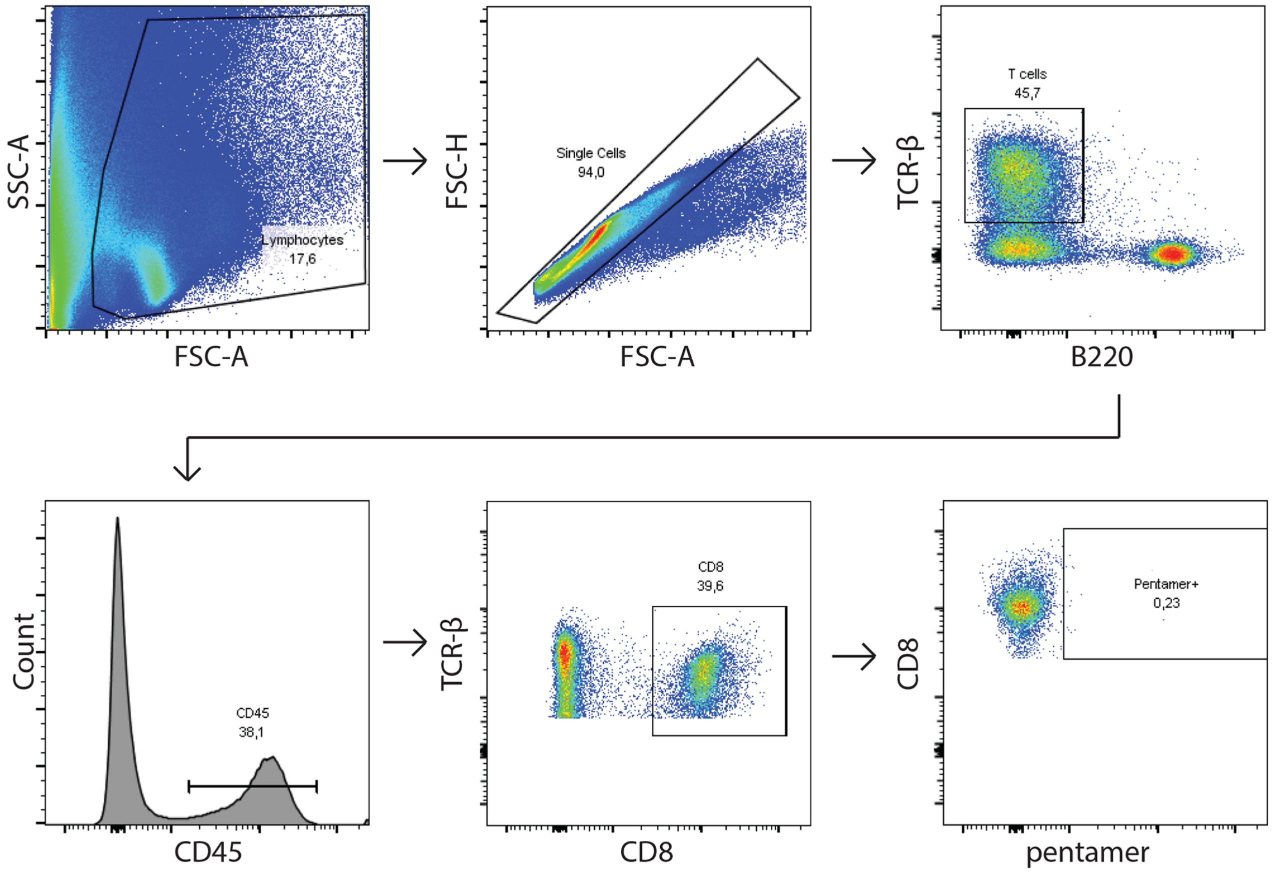

Supplementary Figure S4: Gating strategy for flow cytometry for analysing OVA-specific T cells in the tumour. First lymphocytes were gated on FSC-A and SSC-A, singles were defined by FSC-A and FSC-H, to determine T cells single cells were gated on TCRβ and CD8 positive cells, OVA specific CD8 T cells were defined to be CD8 positive and pentamer positive.

Supplementary Table S1: List of antibodies used in the study.

See Supplementary File 1
