# Supplementary material for: Exosomal cancer immunotherapy is independent of MHC molecules on exosomes
Source: Oncotarget. 2016 May 25;7(25):38707–17. doi: 10.18632/oncotarget.9585 (PMC5122422; doi:10.18632/oncotarget.9585)
Supplement: Supplementary file 2 [file oncotarget-07-38707-s002.docx]

**Supplementary Table 1**

List of antibodies used in the study

| **Antigen** | **Conjugate** | **Clone** | **Company** |
| --- | --- | --- | --- |
| CD1d | PE | 1B1 | Biolegend |
| CD3 | PE/Cy7, Brilliant Violet 605 | 145-2C11 | BDBiosciences |
| CD4 | APC/Cy7, PE | RM4-5 | Biolegend |
| CD8a | Brilliant Violet 605, FITC | 53-6.7 | Biolegend |
| CD9 | PE | MZ3 | Biolegend |
| CD9 | unconjugated | KMC8 | BD Pharmingen |
| CD11c | FITC | HL3 | BDBiosciences |
| CD11c | Pacific Blue | N418 | Biolegend |
| CD11b | Brilliant Violet 785 | M1/70 | Biolegend |
| CD21 | Perc PCy5.5 | 7E9 | Biolegend |
| CD23 | PE | B3B4 | Biolegend |
| CD16/CD32 | unconjugated | 2.4G2 | BDBiosciences |
| CD40 | PE | 3/23 | BDBiosciences |
| CD45 | PercP Cy5.5, APC/Cy7 | 30-F11 | BDBiosciences |
| CD54 | PE | 3E2 | BDBiosciences |
| CD63 | PE | NVG-2 | Biolegend |
| CD80 | PE | 16-10A1 | BDBiosciences |
| CD81 | PE | Eat2 | BDBiosciences |
| CD86 | PE | GL1 | BDBiosciences |
| CD95 | PE-Cy7 | Jo2 | BDBiosciences |
| CD138 | APC | 281-2 | Biolegend |
| BrdU | FITC | 3D4 | BDBiosciences |
| B220 | APC, PE/Cy7, PercpCy5.5, Pacific Blue, FITC | RA3-6B2 | Biolegend |
| B220 | Brilliant Violet 605 | RA3-6B2 | BDBiosciences |
| CXCR5 | PE/Cy7 | 2G8 | BDBiosciences |
| DimerX | unconjugated | N/A | BDBiosciences |
| F4/80 | APC/Cy7 | BM8 | Biolegend |
| GL7 (Ly77) | Pacific Blue | GL7 | Biolegend |
| H2Kb | PE | AF6-88.5 | Biolegend |
| IFNγ | PE/Cy7, PE/Cy7 | XMG1.2 | Biolegend |
| I-A/I-E | PE, APC | M5/114.15.2 | Biolegend |
| Kb/SIINFEKL | PE | eBio25-D1.16 | ebioscience |
| Ly6C | PE | AL-21 | BDBiosciences |
| Ly6G | PE/Cy7 | 1A8 | Biolegend |
| NK1.1 | APC-Cy7, PercP Cy5.5 | PK136 | Biolegend |
| OVA | N/A | 3G2E1D9 | Nordic Biosite |
| OVA-Pentamer | PE | N/A | ProImmune |
| PD-1 | APC | 29F.1A12 | Biolegend |
| TCR-β | Pacific Blue | H57-597 | Biolegend |
| mouse IgG | HRP ELISA | N/A | Southern Biotech |
| mouse IgG1 | Alexa 647 | N/A | Invitrogen |
| rat IgG1 | PE | G0114F7 | Biolegend |
| rat IgG2a | PE | RTK2758 | Biolegend |
| rat IgG2b | PE, FITC | RTK4530 | Biolegend |
| ham IgG1 | PE, FITC | HTK888 | Biolegend |
| mouse IgG1 | PE | MOPC-21 | Biolegend |
| mouse IgG2a | PE | MOPC-173 | Biolegend |
| rat IgG1 | FITC | R3-34 | BDBiosciences |
